# Survival and Death Strategies in Glioma Cells: Autophagy, Senescence and Apoptosis Triggered by a Single Type of Temozolomide-Induced DNA Damage

**DOI:** 10.1371/journal.pone.0055665

**Published:** 2013-01-30

**Authors:** Anna V. Knizhnik, Wynand P. Roos, Teodora Nikolova, Steve Quiros, Karl-Heinz Tomaszowski, Markus Christmann, Bernd Kaina

**Affiliations:** Department of Toxicology, Medical University Center, Mainz, Germany; University Medical Center Hamburg-Eppendorf, Germany

## Abstract

Apoptosis, autophagy, necrosis and cellular senescence are key responses of cells that were exposed to genotoxicants. The types of DNA damage triggering these responses and their interrelationship are largely unknown. Here we studied these responses in glioma cells treated with the methylating agent temozolomide (TMZ), which is a first-line chemotherapeutic for this malignancy. We show that upon TMZ treatment cells undergo autophagy, senescence and apoptosis in a specific time-dependent manner. Necrosis was only marginally induced. All these effects were completely abrogated in isogenic glioma cells expressing O^6^-methylguanine-DNA methyltransferase (MGMT), indicating that a single type of DNA lesion, O^6^-methylguanine (O^6^MeG), is able to trigger all these responses. Studies with mismatch repair mutants and MSH6, Rad51 and ATM knockdowns revealed that autophagy induced by O^6^MeG requires mismatch repair and ATM, and is counteracted by homologous recombination. We further show that autophagy, which precedes apoptosis, is a survival mechanism as its inhibition greatly ameliorated the level of apoptosis following TMZ at therapeutically relevant doses (<100 µM). Cellular senescence increases with post-exposure time and, similar to autophagy, precedes apoptosis. If autophagy was abrogated, TMZ-induced senescence was reduced. Therefore, we propose that autophagy triggered by O^6^MeG adducts is a survival mechanism that stimulates cells to undergo senescence rather than apoptosis. Overall, the data revealed that a specific DNA adduct, O^6^MeG, has the capability of triggering autophagy, senescence and apoptosis and that the decision between survival and death is determined by the balance of players involved. The data also suggests that inhibition of autophagy may ameliorate the therapeutic outcome of TMZ-based cancer therapy.

## Introduction

Astrocytoma and glioblastoma multiforme (GBM) WHO grade III and IV, respectively, are the most common and aggressive malignant primary brain tumors in humans. Radiotherapy is central to the treatment and is often combined with chemotherapy, with temozolomide (TMZ) being the first line chemotherapeutic drug [1]. TMZ induces several DNA adducts among which the minor adduct O^6^-methylguanine (O^6^MeG) is the most cytotoxic lesion, if not repaired by O^6^-methylguanine-DNA methyltransferase (MGMT) [2]. O^6^MeG mispairs with thymine and the resulting O^6^MeG/T mismatches are recognized by the mismatch repair system (MMR), which performs futile repair cycles [3], [4]. During this erroneous repair process secondary lesions (very likely extended gaps) are formed, which block DNA replication in the next replication cycle, leading to DNA double-strand breaks (DSBs) [5], [6]. DSBs eventually signal to apoptosis and possibly other cell death or survival pathways.

It has previously been shown that TMZ induces apoptosis in glioma cells [7]. Kanzawa et al. showed that TMZ also induces autophagy in glioma cell lines and suggested that it was responsible for the TMZ-induced cytotoxicity [8]. There are three different types of autophagic mechanisms: microautophagy, chaperone-mediated autophagy, and macroautophagy (hereafter named autophagy). Autophagy is a process induced by nutrient limitation and cellular stress, which governs degradation of long-lived proteins and whole organelles, thereby maintaining a balance between synthesis, degradation and recycling of cellular components. Autophagy is a highly regulated process and can be activated in response to various stimuli including conditions of starvation, hypoxia, pathogens, radiation, toxic agents and DNA damage (for review see [9]). Autophagy begins with the formation of an isolation membrane (phagophore), which elongates to engulf cytoplasmic components and closes to form an autophagosome. Afterwards the outer membrane of the autophagosome fuses with the lysosome and an autolysosome is formed. The lysosomal hydrolyses degrade the intra-autophagosomal components [10]. Autophagy is regarded both as a cell survival and death mechanism, which depends on the cellular context and treatment conditions. It is also known that autophagy and apoptosis can stimulate or inhibit each other, as they have many players such as Atg5, Bcl-2 and p53 in common. Furthermore, caspases, which are activated during apoptosis, can cleave many autophagy-related proteins and thereby inhibit this process [11].

Another cellular response to chemotherapy following DNA damage is “premature senescence”, which is an inducible form of cellular senescence that is morphologically and biochemically highly related to replicative senescence. Senescent cells are viable, they stop synthesizing DNA, acquire characteristic morphological changes and have increased senescence-associated β-galactosidase activity (SA-β-gal) at an acidic pH [12]. It was shown that TMZ induces cellular senescence in glioma cells cultured as multicellular spheroids [13]. Whether these different endpoints related to survival and death are induced by the same or different DNA damages is an intriguing question that remains to be solved. Also, the interrelation of these key processes triggered by anticancer drugs, including TMZ, is unclear.

In this study we addressed these issues using a glioma cell model and the methylating anticancer drug TMZ, which is being used first-line in the therapy of gliomas and malignant melanomas. The adducts induced by TMZ in the DNA are described [1]. We show that the minor DNA lesion O^6^MeG induced by TMZ triggers all these responses that are evoked in a specific time course in glioma cells. Triggered by O^6^MeG, autophagy induction requires MMR and ataxia telangiectasia mutated protein (ATM), and is reduced by homologous recombination (HR). It serves as a survival mechanism as its inhibition leads to an increased apoptotic response following TMZ treatment. Furthermore we show that TMZ induces senescence in glioma cells, which appears to be a major response to TMZ treatment. Inhibition of autophagy can prevent senescence induction after TMZ. Therefore we propose that autophagy after TMZ treatment serves as a survival mechanism, stimulating cells to undergo senescence and inhibiting apoptosis. If autophagy is abrogated, cells become sensitized to TMZ-induced DNA damage undergoing apoptosis while cellular senescence is blocked. The data revealed that a single type of DNA adduct, O^6^MeG, has the capability to trigger within the same dose range three endpoints that are related to survival and death, namely autophagy, senescence and apoptosis, and the decision between them occurs in a specific temporal manner. In this context, autophagy plays a role of a switch, as it activates senescence and inhibits apoptosis.

## Results

### Temozolomide induces autophagy in glioma cells

**Figure 1:**
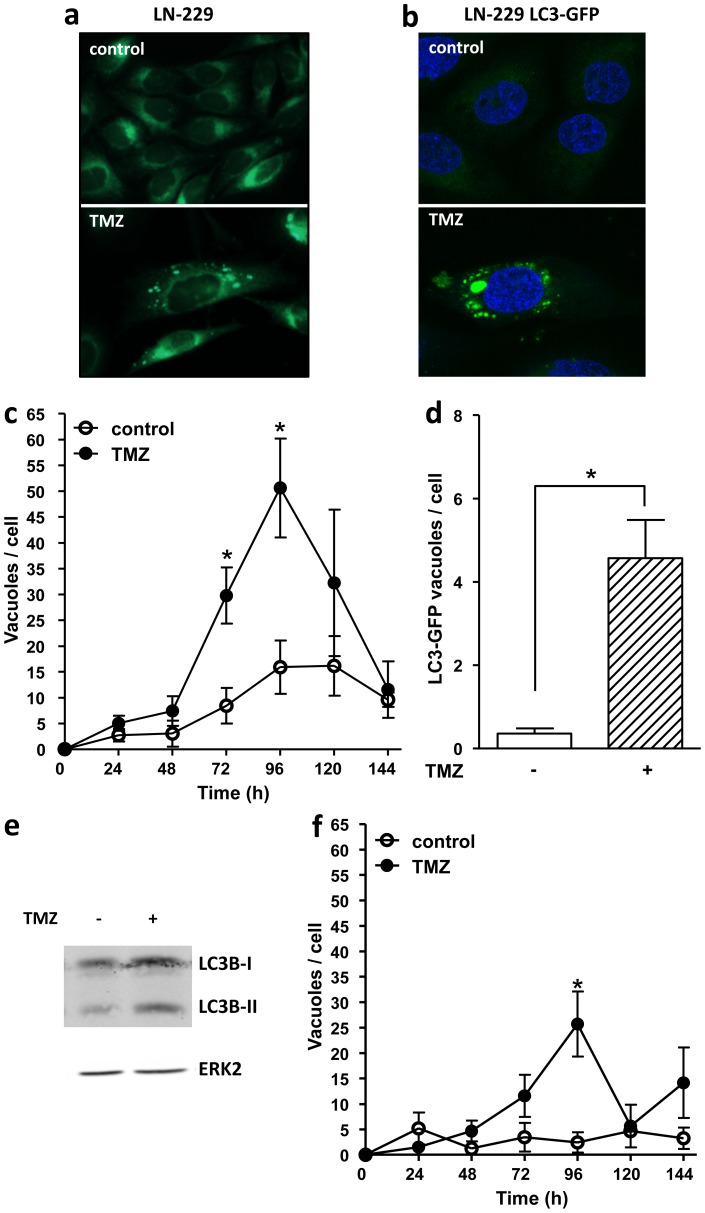
TMZ induces autophagy in LN-229 cells. (**a**) MDC staining of autophagic vacuoles 96 h after TMZ treatment (100 µM)and (**b**) LC3-GFP vacuoles in LN-229 cells after TMZ treatment (100 µM), TO-PRO-3 was used for nuclear staining. (**c**) Quantification of MDC positive vacuoles 24–144 h after TMZ treatment in LN-229 cells. (**d**) Quantification of LC3-GFP positive vacuoles 96 h after TMZ treatment in LN-229 cells. (**e**) LC3B expression 96 h after TMZ treatment (100 µM) performed by western blot, ERK-2 was used as loading control. (**f**) Quantification of MDC positive vacuoles 24–144 h after TMZ treatment (100 µM) in U87 MG cells.

To assess whether TMZ induces autophagy in glioma cells, LN-229 (a glioblastoma line) and U87 MG (an astrocytoma line) were treated with 100 µM TMZ and stained with monodansylcadaverine (MDC) 24–144 h after TMZ exposure (cells were pretreated with O^6^BG in order to deplete residual MGMT activity). MDC is an auto-fluorescent compound, which accumulates in autophagic vacuoles. MDC stained vacuoles were visualized (for a representative example see Fig. 1a) and the rate of autophagy was measured as the average number of MDC stained vacuoles per cell. Significant autophagy (compared to the non-treated control) was detected in LN-229 cells earliest 72 h and a maximum was achieved at 96 h after TMZ treatment. The autophagy rate decreased at later times (Fig. 1c). The lines LN-229 and U87 MG slightly differed in their response as U87 MG cells showed a lower autophagy rate at its maximum, i.e. 96 h after treatment (Fig. 1f). For the majority of the following studies LN-229 was chosen, which is a widely accepted model glioma line. We confirmed autophagy induction by stably expressing LC3-GFP in LN-229 cells. LC3-GFP positive vacuoles serving as proof of autophagy were clearly observed 96 h after TMZ treatment (Fig. 1b and 1d for quantification). We also show that TMZ induces an increase in the lipidated form of LC3B (LC3B-II), which is also a generally accepted marker of autophagy, 96 h after treatment (Fig. 1e). The data support the notion that TMZ induces autophagy in glioma cells, which is a late response following treatment.

### Temozolomide-induced autophagy is prevented by MGMT and requires MMR

**Figure 2:**
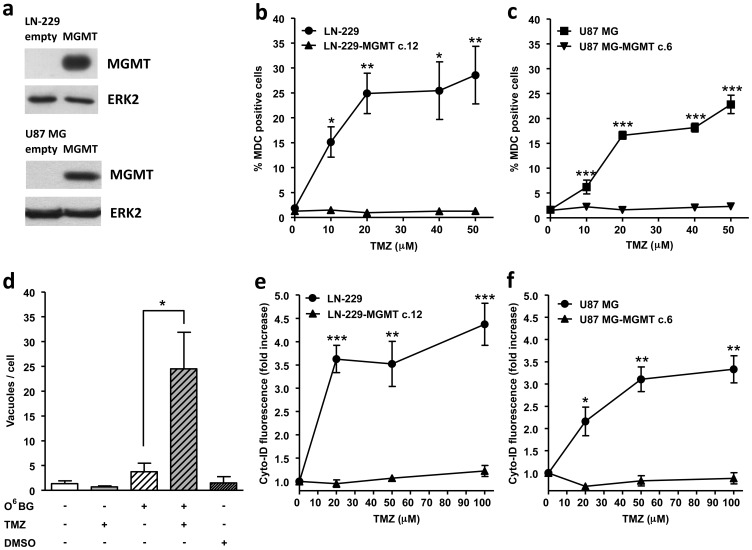
TMZ-induced autophagy is MGMT dependent. (**a**) MGMT expression LN-229 and LN-229 stably transfected with MGMT (LN-229-MGMT c.12) and U87 MG and U87 MG stably transfected with MGMT (U87 MG-MGMT c.6) performed by western blot, ERK-2 was used as loading control. Autophagy induction after TMZ treatment determined by MDC staining and quantified by flow cytometry in (**b**) LN-229 and LN-229-MGMT c.12 and (**c**) U87 MG and U87 MG-MGMT c.6. (**d**) Quantification of MDC positive vacuoles in LN-229-MGMT c.12 cells 96 h after TMZ treatment (100 µM) with or without 1 h pretreatment with 10 µM O^6^BG. Autophagy induction after TMZ treatment determined by Cyto-ID staining and quantified by flow cytometry in (**e**) LN-229 and LN-229-MGMT c.12 and (**f**) U87 MG and U87 MG-MGMT c.6.

TMZ induces 13 DNA adducts among which O^6^MeG is the most cytotoxic lesion when MGMT is depleted [2]. In order to test whether autophagy after TMZ treatment is triggered by this specific DNA adduct, LN-229 and U87 MG cells (both lines do not express detectable amounts of MGMT) were stably transfected with MGMT (Fig. 2a). To quantify MDC stained vacuoles more thoroughly and in high quantities throughout the procedure, we established a flow cytometric method for detection of MDC positive cells (see material and methods). As can be seen from the dose response curves in Fig. 2b and 2c for LN-229 and U87 MG cells respectively, TMZ induces a significant increase in MDC positive cells even at a low dose level of 10 µM, which is only slightly cytotoxic (not shown). Autophagy induction reached a plateau at the dose of >20 µM TMZ with about 30% and 20% of positively stained LN-229 and U87 MG cells, respectively. MGMT expression completely prevented autophagy induction following TMZ treatment (Fig. 2b,c) showing that O^6^MeG is the lesion responsible for inducing autophagy in both cell lines. A reverse experiment was carried out to confirm this finding: MGMT expressing cells (LN-229 MGMT c.12) were treated with O^6^BG to inhibit MGMT and the number of MDC stained vacuoles per cell was counted 96 h following treatment. Inhibition of MGMT activity leads to significant autophagy induction after TMZ treatment (Fig. 2d).

To confirm autophagy induction after TMZ treatment and its dependence on MGMT we performed staining of LN-229 and U87 MG and the corresponding MGMT transfected lines with the Cyto-ID Green Detection Reagent. Cyto-ID serves as a selective marker of autolysosomes and early autophagic compartments. As shown in Fig. 2e and f, TMZ treatment induces an increase in Cyto-ID fluorescence 96 h after treatment in both cell lines and this effect is completely abolished by MGMT expression.

**Figure 3:**
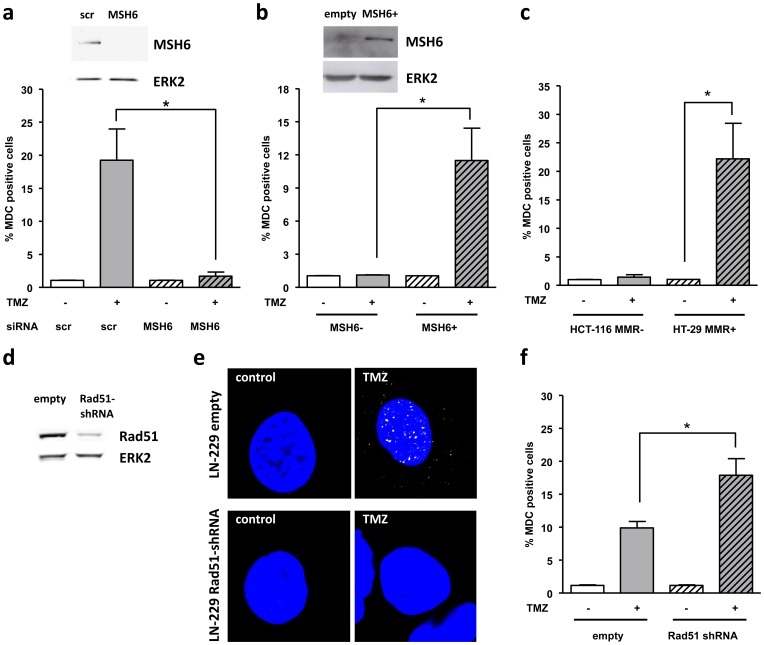
TMZ-induced autophagy is MMR and HR dependent. (**a**) Autophagy induction after TMZ treatment determined by MDC staining and quantified by flow cytometry in LN-229 cells transiently knocked-down for MSH6 (or transfected with scrambled siRNA). Insert: MSH6 expression in LN-229 cells transiently transfected with MSH6 or scrambled siRNA 96 h after transfection performed by western blot, ERK-2 was used as loading control. (**b**) MDC positive cells determined 96 h after TMZ treatment (100 µM) via flow cytometry in stable MSH6 expressing cells (DLD-1-MSH6) compared to control cells. Insert: MSH6 expression in overexpressing clone and control performed by western blot, ERK-2 was used as loading control. (**c**) MDC positive cells in HCT-116 and HT-29 cells, as determined 96 h after TMZ treatment. (**d**) Rad51 expression in stable Rad51 knockdown glioma cells (LN-229 Rad51shRNA) compared to empty vector transfected cells (LN-229-pS-empty) performed by western blot, ERK-2 was used as loading control. (**e**) Rad51 foci (yellow) 72 h after TMZ treatment (20 µM) in knockdown clone and control. TO-PRO-3 was used for nuclear staining (**f**) MDC positive cells determined 96 h after TMZ treatment (10 µM) via flow cytometry in knockdown clone and control.

If not repaired by MGMT, the O^6^MeG/T mismatch is recognized by the MMR system that performs erroneous processing of the damage, which is thought to be central for mediating cell death [14]. To test whether MMR is involved in autophagy induction following TMZ treatment, LN-229 were transiently transfected with MSH6 siRNA. MSH6 is part of the MSH2–MSH6 (MutSα) complex of MMR that recognizes and binds to O^6^MeG/T mismatches. Autophagy induction was monitored using flow cytometry of MDC positive cells. As shown in Fig. 3a, autophagy was completely abrogated by MSH6 downregulation. To verify this finding, a colorectal adenocarcinoma cell line, DLD-1, which is deficient in MSH6, was chosen. MSH6 was expressed in DLD-1 cells and autophagy induction was monitored. As shown in Fig. 3b, no autophagy induction was observed in DLD-1 cells after TMZ treatment, whereas re-expression of MSH6 restores the ability of DLD-1 cells to undergo autophagy after TMZ treatment, thus confirming that active MMR is required for TMZ-induced autophagy. Further proof for this finding was gained by performing the same experiment with two other colon adenocarcinoma cell lines with different MMR status: HT-29 (MMR-proficient) and HCT-116 (MMR-deficient, hMLH1 mt). Autophagy induction after TMZ treatment was only seen in HT-29 cells proficient in MMR (Fig. 3c). The data indicate that MMR on O^6^MeG lesions is involved in triggering autophagy.

### HR protects against temozolomide-induced autophagy

During futile repair cycles of MMR on O^6^MeG/T lesions long gaps of single stranded DNA are formed [4] that block DNA replication in the next replication cycle leading to DSBs, which were proposed to be the downstream cytotoxic lesions inducing the DNA damage response (DDR) pathway and apoptosis [6], [15]. If O^6^MeG generated DSBs trigger autophagy, repair of DSBs are expected to have an impact on this endpoint. To modulate DSB repair following TMZ treatment, LN-229 cells were stably transfected with shRNA against Rad51 (Fig. 3d), a key component of HR that protects against O^6^MeG-triggered cell death [5]. As shown in Fig. 3e, TMZ treatment leads to Rad51 foci formation in the control clone, whereas there are no foci observed in the cells stably transfected with Rad51 shRNA. This confirms a previous finding [16] that Rad51-mediated HR processes occur following TMZ treatment. The light cytoplasmic staining of Rad51 foci after TMZ treatment might be explained by an interaction of Rad51 with mitochondrial DNA following DNA damaging exposures [17]. Rad51 downregulation leads to a significant increase in autophagy induction after TMZ treatment (Fig. 3f). These data suggests that HR protects against autophagy after TMZ treatment and DSBs that are formed as a result of O6MeG processing are crucial for autophagy induction.

### Temozolomide-induced autophagy antagonizes apoptosis

**Figure 4:**
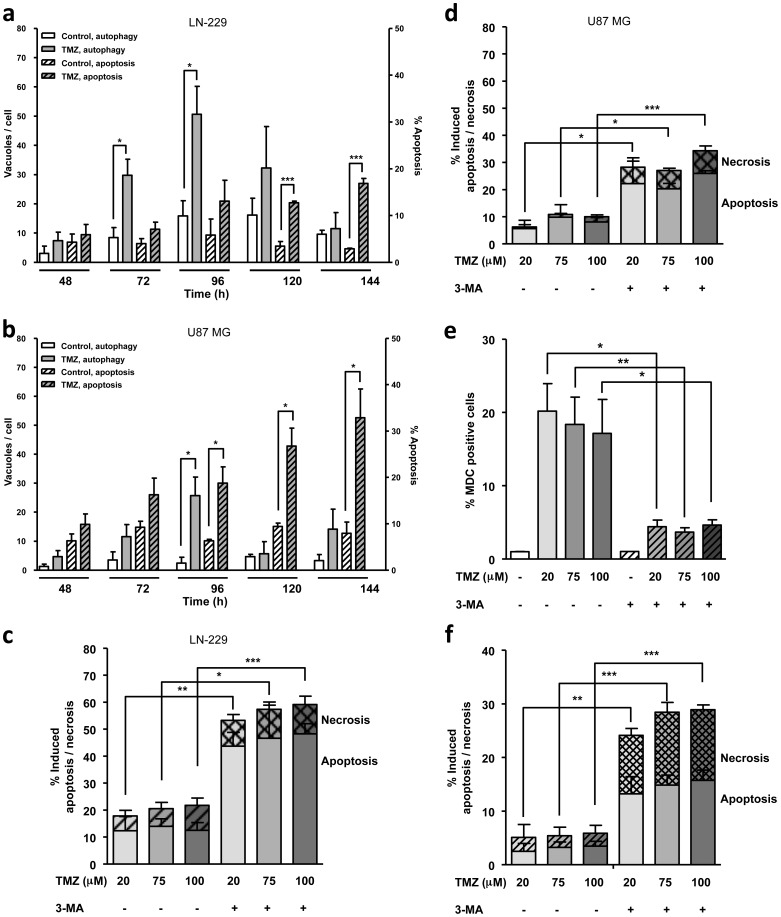
TMZ-induced autophagy comes earlier than apoptosis and inhibits it. Autophagy and apoptosis as determined by MDC staining and Sub-G1 flow cytometry 48–144 h after TMZ treatment (100 µM) in (**a**) LN-229 and (**b**) U87 MG cells. Apoptosis and necrosis was determined by Annexin V-FITC/PI double staining and quantified by flow cytometry 120 h after TMZ treatment in the absence or presence of 3-methyladenine (3-MA, 5 mM, 24 h after TMZ treatment) in (**c**) LN-229 and (**d**) U87 MG cells. (**e**) Autophagy determined by MDC staining and quantified via flow cytometry 96 h after TMZ treatment (100 µM) in LN-229 cells in the absence or presence of 3-MA (5 mM, 24 h after TMZ treatment). (**f**) Apoptosis and necrosis determined by Annexin V-FITC/PI double staining and quantified by flow cytometry 96 h after TMZ treatment in the absence or presence of 3-methyladenine (3-MA, 5 mM, 24 h after TMZ treatment) in LN-229 cells.

In previous studies we showed that TMZ induces apoptosis in glioma cell lines at late times after treatment and that O^6^MeG is the lesion responsible for apoptosis induction [7]. Comparing the time course of apoptosis and autophagy following TMZ treatment it becomes obvious that autophagy precedes apoptosis. Thus autophagy can be detected 72 h after TMZ treatment whereas apoptosis is induced later, from 120 h onwards, in LN-229 cells (Fig. 4a). Although U87 MG cells have a lower autophagy response, the time course is similar with autophagy preceding apoptosis (Fig. 4b). To elucidate the possible interplay between autophagy and apoptosis, LN-229 and U87 MG cells were treated with an early stage autophagy inhibitor 3-methyladenine (3-MA) [18] 24 h after TMZ treatment. As shown in Fig. 4c and 4d, 3-MA treatment leads to a significant increase in the apoptotic fraction, independent of the TMZ concentration used. 3-MA treatment did not show any toxic effect on its own (data not shown). The effectiveness of 3-MA to inhibit autophagy was tested using MDC staining. The data shows that 3-MA was very effective in blocking TMZ-induced autophagy (Fig. 4e). These results support the hypothesis that autophagy serves as a protective and pro-survival mechanism, as demonstrated in LN-229 and U87 MG cells after TMZ treatment, i.e. autophagy inhibits TMZ-induced apoptosis. It is worth noting that when autophagy is inhibited, apoptosis occurs earlier. Its induction in LN-229 cells can already be seen 96 h after TMZ treatment. In addition, significantly higher levels of necrosis (defined as PI and annexin V positive cells) can be seen after TMZ and 3-MA treatment (Fig. 4f), indicating that autophagy also antagonizes necrosis induction.

### ATM is involved in TMZ-induced autophagy

**Figure 5:**
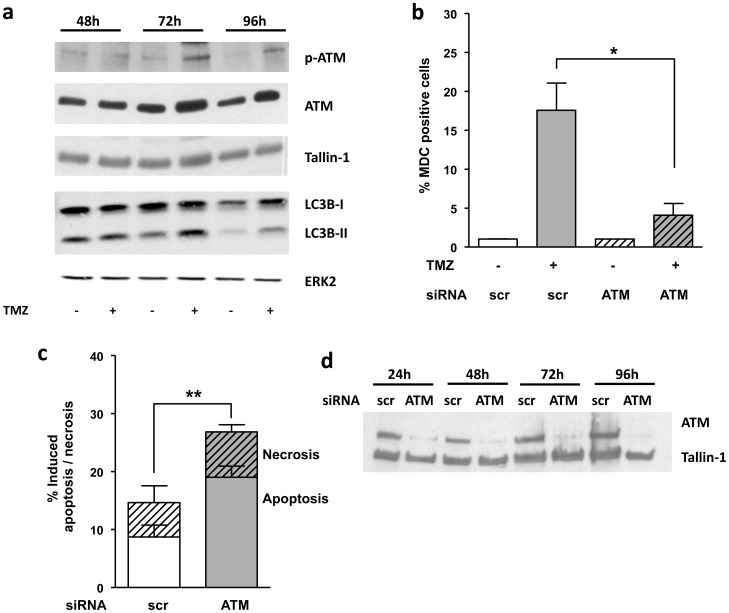
TMZ induces autophagy through activation of ATM. (**a**) Western blot analysis of p-ATM (Ser-1981), ATM and LC3B in LN-229 cells 48–96 h after TMZ treatment (20 µM and 100 µM correspondingly) Tallin-1 and ERK-2 were used for loading control. (**b**) Autophagy positive cells as determined by MDC staining 96 h after TMZ treatment and (**c**) apoptosis and necrosis as determined by Annexin V-FITC/PI double staining 120 h after treatment and quantified by flow cytometry in LN-229 cells transiently knocked-down for ATM (or transfected with scrambled siRNA) treated with TMZ (100 µM). (**d**) Western blot analysis of ATM protein levels of LN-229 cells transiently knocked-down for ATM (or transfected with scrambled siRNA) 24–96 h after transfection. Tallin-1 was used as loading control.

To further investigate the role of DSBs in O^6^MeG-triggered autophagy, we looked at the ATM protein kinase, which is recruited to DSBs via the Mre11-RAD50-NBS1 (MRN) complex and activates signal transduction pathways essential for coordinating cell cycle progression with DNA repair. Phosphorylation of ATM at Ser-1981 occurred 72 h after TMZ treatment, concomitantly with increase in LC3B-II (Fig. 5a). Interestingly, autophagy was completely abrogated after TMZ treatment by ATM downregulation, achieved by transfection with ATM siRNA (Fig. 5b,d). This shows that ATM is required for autophagy induction following DNA damage by TMZ. ATM inhibition leads to an increase in apoptosis (Fig. 5c). Obviously, ATM is required for autophagy induction after TMZ treatment, which antagonizes apoptosis, while ATM inhibition sensitizes LN-229 cells to TMZ.

### O^6^MeG-triggered senescence requires autophagy

**Figure 6:**
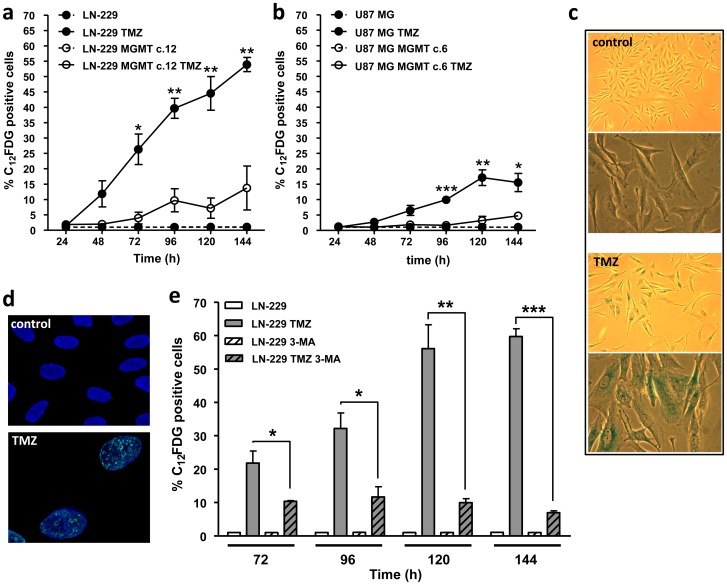
TMZ induces senescence in LN-229 cells, which is dependent on autophagy. Senescence induction 24–144 h after TMZ treatment (100 µM) determined by C_12_FDG positive cells staining and quantified by flow cytometry in (**a**) LN-229 andLN-229-MGMT c.12 and (**b**) U87 MG and U87 MG-MGMT c.6. (**c**) SA-β-gal staining of LN-229 cells 144 h after TMZ treatment. (**d**) Immunofluorescence staining of Histone 3 (tri methyl K9) 144 h after TMZ treatment in LN-229 cells. TO-PRO-3 nuclear staining. (**e**) Senescence induction 72–144 h after TMZ treatment determined by C_12_FDG positive cells staining and quantified by flow cytometry in the absence or presence of 3-MA (5 mM, 24 h after TMZ treatment).

The data shown above demonstrate that a fraction of TMZ treated cells undergoes autophagy and later on this or another fraction undergoes apoptosis. What happens with the damaged cells that do not undergo apoptosis after TMZ treatment? Is cellular senescence triggered as well? Senescent cells express β-galactosidase (β-gal) activity that is detectable at pH 6.0, which is a generally accepted marker designated as senescence-associated β-galactosidase activity (SA-β-gal) [19]. To determine senescence we used 5-dodecanoylaminofluorescein-di-β-D-galactopyranoside (C_12_FDG), a fluorogenic substrate that is cleaved by ß-gal, producing a fluorescent product that is well retained by the cells. Cells were stained with C_12_FDG under conditions of increased lysosomal pH (bafilomycin A1), in order to ascertain the lysosomal origin of SA-β-gal activity. Acidic β-gal activity is present in all cells and can be detected at pH 4.0. As shown in Fig. 6a,b, TMZ is able to induce senescence in LN-229 and, to a lesser extent, in U87 MG cells. Senescence starts to become detectable 48 and 72 h after TMZ addition in LN-229 and U87 MG cells, respectively, and persisted over the whole post-incubation time period (up to 144 h). We also performed a cytochemical senescence detection assay using the chromogenic substrate 5-bromo-4-chloro-3-indoyl β-D-galactopyranoside (X-gal), which yields an insoluble blue compound when cleaved by β-gal (Fig. 6c). Further, we used another marker for senescence, the senescence-associated heterochromatic foci (SAHF), which are usually enriched for histone H3 methylated on lysine 9 (H3K9), which were not observed in non-treated but induced in TMZ-treated cells (Fig. 6d). These assays confirmed that TMZ induces senescence in glioma cells.

Does O^6^MeG trigger senescence? Again, we made use of the isogenic cell model and compared the “wild-type” with LN-229 and U87 MG cells stably transfected with MGMT. Senescence was nearly completely abrogated if MGMT was expressed (Fig. 6a,b). This indicates that O^6^MeG is the key DNA damage that triggers senescence.

It was recently shown that autophagy is induced in senescent cells and is even required for senescence [20]. To test whether autophagy impacts on senescence in our glioma model, we treated LN-229 cells with 3-MA 24 h after TMZ treatment and measured senescence 72–144 h after treatment. The data shown in Fig. 6e revealed that 3-MA completely abolished senescence after TMZ treatment. This supports the notion that autophagy is required for senescence, both of which are triggered by the DNA damage O^6^MeG.

## Discussion

In this study, we demonstrate for the first time that a single type of TMZ-induced DNA lesion, O^6^MeG, triggers three different endpoints in glioma cells: apoptosis, autophagy and senescence. It also triggers necrosis, but necrotic cells were induced only at low level (< 5% of overall cell death) indicating that O^6^MeG is not a major necrotic lesion. All effects were completely abrogated by the expression of MGMT, which indicates that other DNA lesions induced by TMZ do not play a significant role in triggering these responses. We should stress the point that we used TMZ concentrations of ≤100 µM, which is in the range of the plasma level (30–80 µM) achieved during chemotherapy [21], [22]. We further show that the endpoints have a complex interplay and that the decision between death and survival depends on a balance of autophagy, senescence and apoptosis.

The time course of autophagy, senescence and apoptosis induced by O^6^MeG is worth discussing. Autophagy induction starts to become significant 72 h after treatment, reaches a maximum at 96 h and decreases thereafter; cellular senescence is induced 72 h after treatment and steadily increases with time, while apoptosis is the latest response becoming significant 120 h after treatment in LN-229 glioblastoma cells. As autophagy and senescence precede apoptosis, their modulation might have an impact on the level of cell death, which was indeed the case.

**Figure 7:**
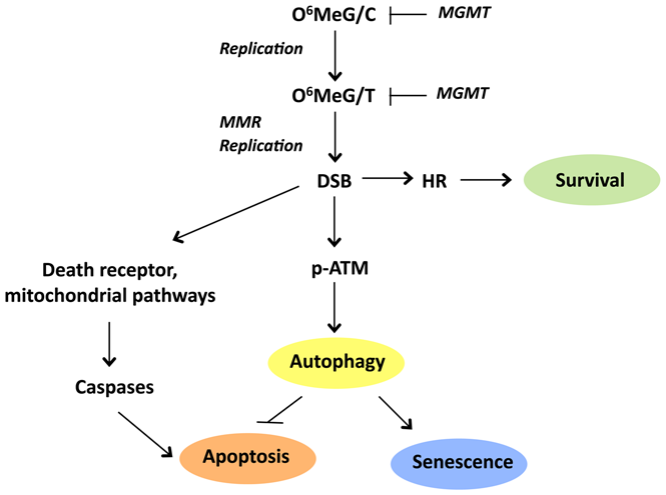
Pathway of apoptosis, autophagy, and senescence triggered by O^6^MeG, which is induced in the DNA by TMZ and other O^6^-alkylating anticancer drugs. O^6^MeG is converted via replication and MMR into DSBs that trigger autophagy and senescence for which ATM is required. O^6^MeG mediated DSBs also trigger caspase-dependent apoptosis, which is attenuated by autophagy and ameliorated if autophagy is blocked. Inhibition of autophagy following TMZ also leads to attenuation of senescence. The major pathway of repair of O^6^MeG-induced DSBs is HR, leading to protection against both autophagy, senescence and apoptosis.

It is well established that futile MMR is required for O^6^MeG triggered apoptosis by converting O^6^MeG/T mispairs into secondary lesions [3], [23], [24]. Therefore, we wondered whether MMR is also needed for O^6^MeG triggered autophagy. In MMR defective cells autophagy was not observed following TMZ, suggesting that MMR of O^6^MeG/T mispairs is critically involved. Furthermore, downregulation of Rad51 by stable shRNA transfection that attenuates HR, which is the major pathway for repairing DSBs in response to TMZ [25], [26], significantly ameliorated autophagy, indicating that DSBs formed in response to O^6^MeG/T are the critical downstream DNA lesion. On the basis of these data, we conclude that autophagy is triggered by the same upstream DNA damage pathway (see Fig. 7 for a model) that is involved in triggering apoptosis [2], [7], [14]. Since two DNA replication cycles are required for DSBs to be formed after O^6^MeG lesion induction, as described previously [5], autophagy is expected to be a late response, which is indeed the case, as it first becomes visible 72 h after treatment.

It was shown that TMZ treatment results in ATM activation, which is the result of O^6^MeG processing [4], [27]. We confirmed this and further demonstrate that TMZ-induced autophagy is ATM dependent as ATM downregulation attenuated the autophagy response. This is in accordance with data obtained with human lymphoblastoid cells showing that knockout of ATM prevents the induction of autophagy in response to reactive oxygen species (ROS) [28]. We also showed that ATM downregulation leads to an increase in the apoptotic fraction after TMZ treatment, which supports the view that ATM is involved in the signaling pathway that regulates autophagy and apoptosis.

What is the biological role of autophagy in glioma cells treated with TMZ? There are reports claiming that autophagy is a pro- and anti-survival mechanism following TMZ. Thus, Kanzawa et al. reported that TMZ induces autophagy and proposed it to be responsible for TMZ-induced cell death, as apoptotic cells were not observed under their settings [8]. We should again stress the point that apoptosis in glioma cells following TMZ is a late response, occurring ≥120 h after induction of O^6^MeG [7], while in the study mentioned above samples were taken at early time points when apoptosis is not yet occurring. It was also reported that the combined administration of TMZ and 3-MA leads to increased cell viability in comparison to TMZ alone [8], which does not correspond to our data. In the present study we show that a combination of TMZ and 3-MA inhibits autophagy and potentiates apoptosis in comparison to treatment with TMZ alone, indicating that autophagy is a survival mechanism. Our findings are in line with the work of Lin et al. who reported that TMZ-induced autophagy protects glioma cells against apoptosis. We should note that in this study high TMZ doses were used (400 µM compared to ≤100 µM used in our study) at which DNA base N-alkylations likely play a major role in eliciting cytotoxicity. In line with this is the finding that the killing effects were observed at quite early time points (72 h) after treatment [29], indicating that O^6^MeG was not the lesion responsible for autophagy.

There is another report showing that autophagy inhibition prevents from cytotoxic effects of ionizing radiation in combination with high dose TMZ [30]. Again, under these conditions other lesions than O^6^MeG might be dominant, shifting the balance of the autophagy pathway from survival to death. In the experiments reported here with two well-characterized glioma lines we demonstrate that a) autophagy precedes apoptosis, b) inhibition of autophagy by 3-MA ameliorates the level of apoptosis, and c) downregulation of ATM inhibits autophagy and ameliorates apoptosis. Altogether the data support the notion that autophagy and apoptosis are interrelated with autophagy protecting against O^6^MeG-triggered cell death. The data are in accordance with the view that autophagy is a universal cytoprotective mechanism against DNA damaging chemotherapeutic agents, which was recently also proposed on the basis of data obtained with hepatoma cell lines [31].

It was previously shown that TMZ induces senescence in glioma cells [13], [32], which is confirmed in this study. Senescence induction is O^6^MeG dependent as MGMT expression abolished it nearly to completion. We further show that inhibition of autophagy prevents from senescence induction after TMZ treatment, indicating that autophagy is directly related to senescence. Our data are in agreement with a study on human diploid fibroblasts showing that autophagy is required for senescence induced by the *HRAS* oncogene [20]. In Fig. 7, we outline our current understanding of the processes occurring after O^6^MeG lesion induction, pointing out that HR protects against autophagy and that autophagy is dependent on MMR and ATM. Autophagy is regarded as a survival mechanism stimulating cells to undergo senescence and, on the other hand, inhibiting apoptosis. We wish to propose that if autophagy is inhibited after TMZ treatment cells no longer undergo senescence and switch to the apoptosis pathway. This notion is supported by the finding that transient activation of autophagy shifts apoptosis towards senescence in a vascular aging model in culture [33]. Furthermore, decrease in DNA-damage induced senescence and increase in apoptosis upon autophagy inhibition was demonstrated [34].

The mechanism of how autophagy regulates senescence is still unclear. It is possible that the phenotypic changes observed during cellular senescence require targeted degradation of specific cellular components. Alternatively, autophagy may provide building blocks for macromolecules synthesis in conditions of energy demand, which is present because senescent cells are metabolically and biosynthetically active [35]. The identification of a specific DNA damage, O^6^MeG, as a common trigger for these endpoints may provide a basis for future experiments designed to elucidate the complex pathways involved.

In conclusion, we demonstrate that TMZ at therapeutic relevant dose levels (≤100 µM) induces autophagy, cellular senescence and apoptosis in glioma cells. This occurs in a sequential manner with senescence and autophagy preceding apoptosis. Since MGMT suppressed all these endpoints nearly to completion, we conclude that they are triggered by the same DNA adduct, O^6^MeG. From inhibitor experiments we further infer that autophagy serves as a survival mechanism inhibiting apoptosis and stimulating senescence. The pharmacological inhibition of autophagy in combination with TMZ treatment might represent a strategy for ameliorating the killing effect of TMZ on glioma cells and thus enhance the therapeutic response.

## Materials and Methods

### Cell lines and culture conditions

LN-229 and U87 MG glioma cell lines were provided by Dr. Weller (Department of Oncology, University Hospital Zurich) and extensively described previously [36]. DLD1 and HCT-116 are previously described colon carcinoma cell lines [37], which were kindly provided by Dr. Kleinert (Institute for Pharmacology, Medical University Center Mainz) and Dr. Issinger (Department of Biochemistry, University of Southern Denmark) respectively. HT-29 was purchased from ATCC. All cell lines, except HT-29, were maintained in Dulbecco's modified Eagle's medium (DMEM) with 10% fetal bovine serum (FBS) and penicillin (100 unit/ml) streptomycin (100 µg/ml) (P/S) in 37°C and 5% CO_2_ atmosphere. HT-29 was cultured in RPMI medium with 10% FBS and P/S. Cells were checked for mycoplasma contamination before experimental use.

### Plasmids and stable transfections

LN-229 and U87 MG cells were stably transfected with MGMT in pSV2MGMT vector and the pSV2neo plasmid for selection; short hairpin RNA (shRNA) targeting Rad51 or with empty vector; pEGFP-C1 vector. DLD-1 cells were transfected with MSH6 in pcDNA3.1 vector. Plasmids were transfected using Effectene (Quiagen). Selection of transfected cells occurred with 1.5 mg/ml G418 (LN-229, U87 MG) and 1 mg/ml G418 (DLD-1) until clones were formed.

### Transient transfections

ATM #M-003201-04, MSH6 #M-019287-01 or Non-targeting #D-001206-13 siRNA pools of four siRNAs (Dharmacon, Thermo Scientific) were transfected with Lipofectamine RNAiMAX (Invitrogen).

### Drugs and drug treatment

TMZ (Schering-Plough) was dissolved in DMSO and sterile dH_2_O (1∶2) to a concentration of 35 mM. O^6^-benzylguanine (O^6^BG, Sigma) was dissolved in DMSO to a concentration of 10 mM. 3-methyladenine (3-MA, Sigma) was dissolved in sterile dH_2_O to a concentration of 200 mM with heating. For MGMT depletion O^6^BG (10 µM) was added 1 h prior to drug treatments. All experiments were repeated at least three times and data were evaluated statistically using the t-test (* p<0.05; ** p<0.01;*** p<0.001).

### Determination of apoptosis and necrosis

The apoptotic frequency was determined by Sub-G1 fraction analysis or Annexin V-FITC/propidium iodide (PI) double-staining and quantified by flow cytometry with FACSCanto (Becton Dickinson) as previously described [38].

### Determination of autophagy (MDC staining)

For flow cytometric analysis of MDC stained cells, harvested cells were stained with 0.1 mM MDC (Sigma) for 30 min at room temperature, washed 3 times with PBS, fixed with 1% formaldehyde at room temperature, washed 3 times with PBS and resuspended in PBS. Cell suspension was immediately quantified by flow cytometry with FACSCanto (Becton Dickinson).

For microscopical MDC staining cells were seeded on cover slips. After treatment with TMZ cells were stained with 0.1 mM MDC for 30 min at room temperature, washed 3 times with PBS, fixed with 1% formaldehyde at room temperature, washed 3 times with PBS and slides were mounted in antifade medium (Glycerol∶PBS 1∶1, 2.5% DABCO, pH 8.6 with HCl) and scored with Carl Zeiss Axiovert 35 microscope. Images were analysed with CellA Software (Soft Imaging System). Each value represents the average fluorescence intensity of 3 experiments, each of 50 cells.

### Determination of autophagy (LC3-GFP)

LN-229 transfected with pEGFP-C1-LC3 were seeded on cover slips. After treatment with TMZ cells were fixed with 4% formaldehyde for 15 min, washed with PBS and nuclei were counterstained with 1 µM TO-PRO-3 for 15 min. Slides were mounted in antifade medium (Glycerol∶PBS 1∶1, 2.5% DABCO, pH 8.6 with HCl). Fluorescence images were recorded and quantified with a laser scanning microscope (LSM 710) and the ZEN Software from Carl Zeiss AG. Each value represents the average fluorescence intensity of 3 experiments, each of 50 cells.

### Determination of autophagy (Cyto-ID staining)

Flow cytometric analysis of Cyto-ID Green Detection Reagent stained cells was performed according to manufacturer's protocol (Cyto-ID Autophagy Detection Kit, ENZ-51031-K200, Enzo Life Sciences). In brief, harvested cells were washed with PBS, stained with Cyto-ID in indicator-free medium with 5% FBS for 30 min at 37°C, washed with 1× Assay Buffer and resuspended in 1× Assay Buffer. Cell suspension was immediately quantified by flow cytometry with FACSCanto (Becton Dickinson).

### Immunofluorescence

Cells were seeded on cover slips. After treatment with TMZ cells were fixed with 4% formaldehyde, washed with PBS, permeabelized in ice-cold methanol for 10 min at −20°C, rehydrated in PBS and blocked/permeabilized with PBS with 5% BSA and 0.25% Triton-X100 for 1 h. Coverslips were incubated with rabbit anti-Rad51 antibody (Abcam) overnight, washed with PBS and incubated with Alexa Fluor 488 anti-rabbit secondary antibody (Invitrogen) for 1 h. Nuclei were counterstained with 1 µM TO-PRO-3 for 15 min. Slides were mounted in antifade medium (Glycerol∶PBS 1∶1, 2.5% DABCO, pH 8.6 with HCl). Fluorescence images were recorded with a laser scanning microscope (LSM 710) and the ZEN Software from Carl Zeiss AG.

### Determination of senescence

Senescence determination using flow cytometry was performed as previously described [39]. Cytochemical senescence detection was performed with the Senescence β-Galactosidase Staining Kit (Cell Signaling). For Histone 3 (tri methyl K9) immunofluorescence staining LN-229 cells were seeded on cover slips. 144 h following treatment with TMZ cells were fixed with Methanol∶Aceton (3∶1) for 7 min. Cover slips were then blocked with 5% bovine serum albumin in PBS containing 0.3% Triton X-100. Incubated with anti-Histone 3 (tri methyl K9) (Abcam) overnight, washed 3 times in PBS 0.3% Triton X-100 for 5 min and stained with Alexa Fluor 488 (Invitrogen) for 2 h, washed 3 times in PBS 0.3% Triton X-100 for 5 minutes. DNA was stained with 1 µmol/L TO-PRO-3 for 15 minutes. Slides were mounted in antifade medium (Glycerol∶PBS 1∶1, 2.5% DABCO, pH 8.6 with HCl). Images were recorded with a laser scanning microscope (LSM 710) and the ZEN Software from Carl Zeiss.

### Preparation of protein extracts and Western-blot analysis

Whole-cell extracts preparation and Western blot analysis were conducted as previously described [5], [40]. Western blot detection was performed with Odyssey 9120 Infrared Imaging System (Li-Cor Biosciences). The following antibodies were used: anti-ERK2 (Santa Cruz Biotechnology); anti-MSH6 (BD Biosciences); anti-Rad51 (PC-130, Calbiochem); anti-phospho-ATM (Ser-1981) (Milipore); anti-ATM (Cell Signaling); anti-Tallin-1 (Cell Signaling); anti-MGMT (Milipore), anti-LC3B (Cell Signaling).
